# Splashing of tungsten-based anode during arc discharge

**DOI:** 10.1038/s41598-023-39274-4

**Published:** 2023-07-27

**Authors:** Kenta Iida, Hisaya Komen, Masaya Shigeta, Manabu Tanaka

**Affiliations:** 1 Joining and Welding Research Institute, Osaka University, Osaka, Japan; 2 Department of Mechanical Systems Engineering, Tohoku University, Sendai, Japan

**Keywords:** Engineering, Materials science

## Abstract

A unique mechanism of splashing from a tungsten-based anode was identified during arc discharge. Splashing occurred by breakoff of a liquid metal column, which elongates after a local concavity formed on the molten anode surface. Blue–violet luminescence, emitted by cerium ions originating from additives in the tungsten-based anode, was captured before the concavity formation. The surface temperature exceeded the boiling point of the additives at the time of splashing. The measured droplet speeds suggested that an electromagnetic force contributes the high-speed ejections. Energy dispersive spectrometry mapping also exhibited a remnant of the additives on the longitudinal cross-section of the anode after arc discharge. Based on these experimental facts, the mechanism of anode splashing in arc discharge was deduced as follows: bubble formation of additives at temperatures above their boiling point, bubble bursting at the surface, micro-plasma jet generation, liquid-column elongation and breakoff under an electromagnetic force, and consequent high-speed droplet ejection.

## Introduction

Electric discharge occurs when an electrical current flows through a conductive gas medium consisting of electrons and ionised species of molecules and atoms. Such a medium is called plasma. An arc discharge is a type of electric discharge caused by low voltage and high current between a cathode and an anode under atmospheric pressure. As arc discharges can produce remarkably high temperatures (> 10,000 K), arc plasma has been applied as a unique heat source in high-speed metal cutting and joining^1–3^. Moreover, it is attracting scientific and industrial attention as a promising tool for three-dimensional material fabrication, known as additive manufacturing^4,5^. Owing to its high brightness, arc plasma is also used as a light source. As photovoltaics are expected as sources of sustainable electricity in the near future, improving the conversion efficiency of sunlight into electricity is a dominant social problem^6,7^. To accurately measure the conversion efficiency, a stable light source with an emission spectrum equivalent to that of sunlight is required. Among the light sources satisfying these requirements are xenon arc lamps and metal halide arc lamps^8–10^. Recently, we identified the unique dynamics of a molten electrode surface interacting with arc plasma. The present paper elaborates on these findings.

A cathode emits thermionic electrons when heated to high temperature. When the arc current is sufficiently supplied by thermionic emission alone, the arc plasma remains relatively stable. For this reason, the cathode material must be solid or liquid even at temperatures above 3000 K. The usual cathode material is tungsten, which has a high melting point and a high boiling point. Moreover, tungsten doped with a few weight percent of oxides has a lower effective work function and lower thermionic emission than pure tungsten. Consequently, the cathode temperature is reduced and cathode erosion is suppressed^11,12^. Furthermore, by considering the diffusion and evaporation of additives in the cathode, researchers have made improved predictions of cathode erosion^13,14^.

Although the phenomena of tungsten-based cathode during arc discharge have been well studied, the phenomena of the tungsten-based anode are largely unexplained. One such phenomenon is splashing of the molten anode. As the arc current can be either direct current (DC) or alternating current (AC)^15–17^, a single tungsten-based electrode becomes both the cathode and anode. However, the physics of tungsten-based anodes are poorly understood, so the electrode phenomena during AC arc discharge (which is more complex than DC arcing) remain unknown. In particular, splashing of the molten anode accelerates the anode erosion and hinders the formation of stable arc plasma. Furthermore, splashing of molten metals causes contamination and reduces the quality of applications such as arc lamps, materials joining, and additive manufacturing. Several studies have also reported that part of a molten cathode is ejected as droplets during DC arc discharge^18,19^. Notably, however, these studies were conducted under specific conditions immediately after arc ignition or arc extinction^18,19^. In contrast, we observed the droplet ejections from a tungsten-based anode during continuous operation of an arc discharge. This study reveals the dynamics and mechanism of the splashing process on the tungsten-based anode surface during a sustained DC arc discharge. These novel insights will benefit both the science and industrial applications of arc discharge.

## Results

### Splashing behaviour of the tungsten-based anode

**Figure 1:**
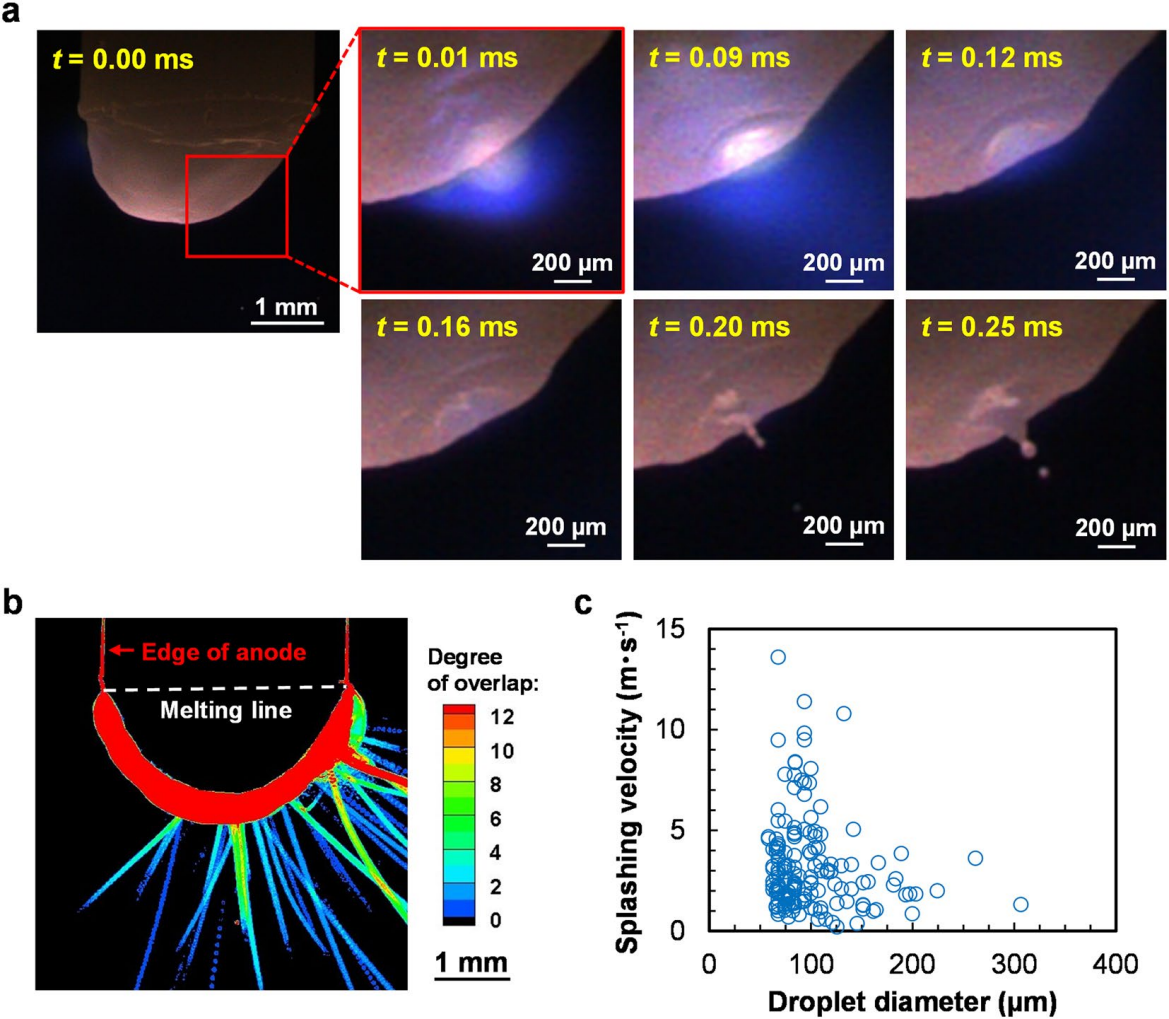
Splashing phenomena from a ceria-doped tungsten anode: (**a**) Droplet emission process captured by a high-speed camera (the video is available in the Supplementary Information); (**b**) trajectories of droplets from the anode; (**c**) Relationship between splashing velocity and droplet diameter.

The splashing phenomena of the anode during arc discharge were visualised using a high-speed camera. The anode material was 2 wt% ceria-doped tungsten and the current was set to 40 A. Figure 1a shows the anode appearance during the arc discharge. The elapsed time *t* is indicated by the yellow characters in the upper left of the images. Here, *t* = 0.00 ms indicates the time at 3 s or longer after arc ignition. At *t* = 0.00 ms, the anode tip was melted and no irregularities or other peculiar shapes appeared on the anode surface. Focusing on the area enclosed by the red frame in the first image, blue–violet light emission appeared near the anode surface at *t* = 0.01 ms. At *t* = 0.20 ms, a cavity with an approximate diameter of 200 µm was formed at the site of light emission. A liquid metal column extended from the centre of the cavity and its tip was split to release a molten metal droplet. A series of splashing phenomena was confirmed on a timescale of ~ 0.3 ms.

Figure 1b shows the splashing trajectories visualised from the anode during 0.1 s. This image was generated through image processing. The colour bar indicates the number of passes of the droplet over the coordinates captured by each pixel during the 0.1-s interval (frame rate = 75,000 fps). The reddest regions indicate where many droplets passed over the position or passed at slow speed. The edge of the anode (also shown in red) was around 0.4 mm wide, indicating that the anode was melted and oscillated during the arc discharge. To highlight the increased edge width in the molten region, the solid–liquid boundary of the anode is shown as a white dashed line. Judging from the visualisation results, splashing occurred from both the anode tip and ~ 1.5 mm above the tip. Figure 1c is a splashing velocity versus diameter plot of many splashed droplets measured over a 1-s interval. The splashing velocities of large droplets (~ 200 µm in diameter) were less than 4 m s^−1^. In contrast, the splashing velocities of droplets with diameters below 100 µm were widely distributed between 0.2 and 14 m s^−1^. The average diameter and velocity of the droplets were 99 µm and 3.2 m s^−1^, respectively.

To clarify the splashing mechanism occurring on the tungsten-based anode during arc discharge, we must identify the driving force of the splashing shown in Fig. 1a. However, to our knowledge, droplet ejection at local concavities on the anode surface has not been reported. Therefore, we investigated why local concavities formed on the anode surface during arc discharge. To this end, we first focused on the blue–violet luminescent zone observed at *t* = 0.01–0.12 ms, which immediately preceded concavity formation. Cerium ions exhibit strong line-emission spectra at wavelengths less than 460 nm^20^, corresponding to the blue–violet colour of the emission area. In a previous experiment, we observed similar blue–violet light emissions around a ceria-doped tungsten cathode after reversing only the electrode polarity^21^. Spectroscopic analysis identified a line-emission spectrum with a wavelength of 456.2 nm originating from cerium ions. This luminescence was caused by the vapour of cerium oxide (an electrode additive), which evaporated and became a plasma^21^. Therefore, the blue–violet luminescent zone in Fig. 1a was inferred as vapour derived from ceria (an additive in the anode). It was rationalised that gasification of ceria largely affects the splash formation at the anode.

Meanwhile, studies on fluid engineering other than electric discharge have shown that cavities form via bubble bursting at gas–liquid interfaces, and that droplets are ejected with the growth of a liquid column^22–24^. Therefore, it was hypothesised that ceria bubbles generated inside the anode are burst at the anode surface. Vapour is ejected with light emissions, forming a cavity on the surface during arc discharge.

### Splashing amount and anode surface temperature

**Figure 2:**
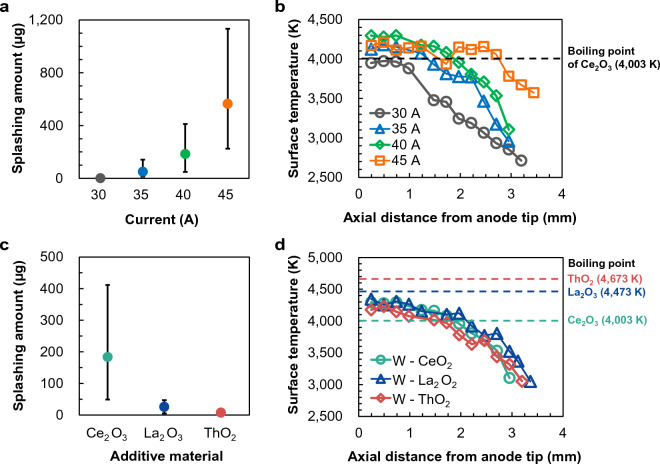
Effect of additive boiling point on the splashing phenomena: (**a**) Splashing amounts; (**b**) axial distributions of surface temperature of the ceria-doped anode at different currents; (**c**) splashing amounts; (**d**) axial distributions of surface temperature of anodes with different additive materials.

To verify our hypothesis, we investigated whether splashing occurred in a region hotter than the boiling point of the additives. To this end, we measured the anode surface temperature. Figure 2a shows the splashing amounts measured over a 0.1-s interval for different current values. Plotted are the average values of five measurements and their error bars (bounded by the maximum and minimum values). No splashing was observed at 30 A. At 35, 40, and 45 A, the splashing amounts were approximately 50, 184, and 565 µg, respectively. Clearly, the splashing amount increased with current. Figure 2b shows the surface temperature distributions along the anode axis for different current values. When the current was set to 30 A, the anode-tip temperature was 3950 K and the surface temperature was below the boiling point of Ce_2_O_3_ (4003 K)^25^. At 35, 40, and 45 A, the anode-tip temperatures were 4120, 4300, and 4171 K, respectively, exceeding the boiling point of Ce_2_O_3_. The temperature ranged above the boiling point of Ce_2_O_3_ at approximately 1.3, 1.8, and 2.8 mm from the anode tip at 35, 40, and 45 A, respectively. These results can explain the increase in splashing amount with current; specifically, increasing the current expanded the area over which the temperature range exceeded the boiling point of ceria.

Similar measurements were conducted on tungsten anodes with different dopants. Figure 2c shows the measured splashing amounts from W–2 wt%Ce_2_O_3_, W–2 wt%La_2_O_3,_ and W–2 wt%ThO_2_ anodes at constant current (40 A). The average splashing amounts were approximately 184, 26, and 7 µg from the ceria-doped, lanthana-doped, and thoria-doped tungsten anodes, respectively. Figure 2d shows the surface temperature distributions along the central axis of the anodes doped with different additives, along with the boiling point of each additive^25–27^. The tip temperatures of the ceria-doped, lanthana-doped, and thoria-doped tungsten anodes were 4300, 4340, and 4180 K, respectively. The surface temperature distributions did not clearly differ among the anodes with different additive materials, although the actual temperatures varied by up to 400 K depending on the additive. The large difference in splashing amounts despite the similar temperature distributions was attributed to the different boiling points of the additives. When the anode was ceria-doped tungsten, the surface temperature exceeded the boiling point of Ce_2_O_3_ (4003 K) up to 1.8 mm above the anode tip. In contrast, the surface temperature of the lanthana-doped tungsten anode was below the boiling point of La_2_O_3_ (4473 K) at all positions (~ 130 K lower at the anode tip). Similarly, the surface temperature of the thoria-doped tungsten anode was below the boiling point of ThO_2_ (4673 K) at all positions (~ 500 K lower at the anode tip). Therefore, the splashing amounts from the lanthana-doped and thoria-doped tungsten anodes were smaller than that from ceria-doped tungsten because their surface temperatures were lower than the boiling points of their respective additives.

### Analysis of anode cross-section

**Figure 3:**
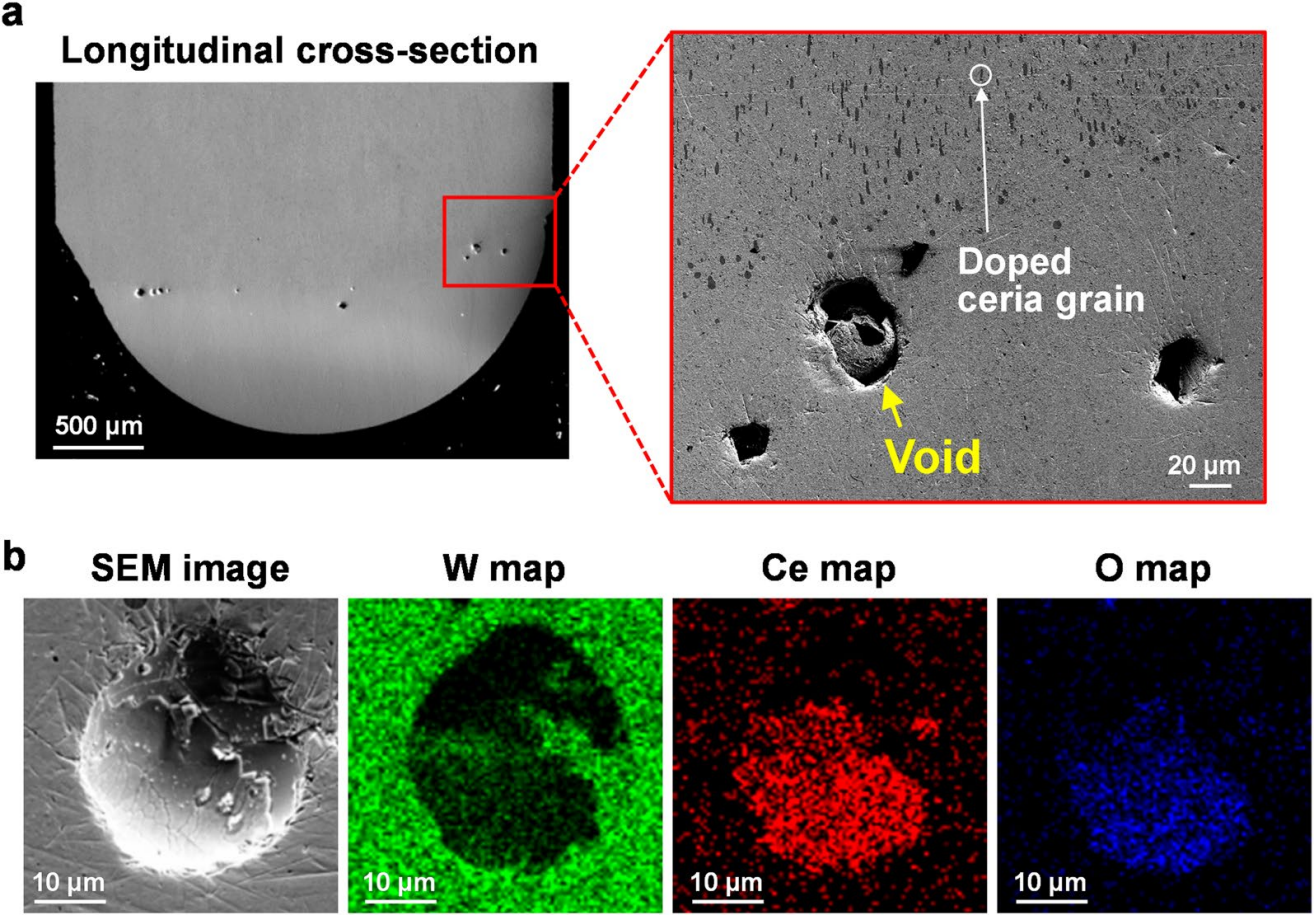
Analysis of the interior of the W–2 wt%Ce_2_O_3_ anode after arc discharge: (**a**) SEM image of the longitudinal cross-section of the anode; (**b**) SEM image and EDS maps of a void inside the anode after arc discharge.

According to the measured splashing amounts and anode surface temperatures, splashing occurred when the anode temperature exceeded the boiling point of the additive in the anode. This suggests that the additive can be gasified inside the anode. To confirm the occurrence or non-occurrence of gasification, the cross-section of the anode after arc discharge was analysed. Figure 3a shows scanning electron microscopy (SEM) images of the longitudinal cross-section of the ceria-doped tungsten anode after three seconds of arc discharge at 40 A. Within the area enclosed by the red frame, multiple voids ~ 40 µm in diameter are visible. Figure 3b shows the energy dispersive X-ray spectroscopy (EDS) mappings of one void. Tungsten, the main anode material, was detected outside the void, but cerium and oxygen were enriched inside the void, suggesting that the void was filled with ceria vapour. Granular ceria with diameters ~ 5 µm were dispersed in the anode before the arc discharge^11^. After the arc discharge, the void diameters were ~ 10 times larger than the original ceria grains. This result clarifies that the ceria grains were gasified and bubbles were formed inside the anode when the anode tip was heated above the boiling point of ceria during the arc discharge.

### Liquid column formation process

The mechanical behaviour after a concavity formation has been discussed in fluid mechanics studies^28–30^. The surface tension acting on the rim of the concavity drives the flow towards the bottom of the concavity, forming a liquid column. Driving forces other than surface tension also act on the anode surface during arc discharge, but the effects of these forces on droplet ejection have not been clarified.

To discuss the liquid-column formation process, we focus on the droplet-ejection velocity. As confirmed in Fig. 1c, the droplet diameter is related to the droplet-ejection velocity during arc discharge. In addition, the droplet-ejection velocity caused by surface tension with bubble bursting is determined as^31^

1 $$v=\sqrt{\frac{2\sigma }{\rho {d}_{\mathrm{b}}},}$$

where $v$ is the droplet-ejection velocity,$\sigma$ is the surface tension of the liquid, $\rho$ is the liquid density, and ${d}_{\mathrm{b}}$ is the diameter of the bubble when bursting at the gas–liquid interface. To investigate the influence of driving forces other than surface tension, the droplet-ejection velocity estimated using Eq. (1) was compared with the experimentally determined splashing velocity.

To estimate the theoretical velocity by Eq. (1), we must know the diameter of the bubble bursting at the anode surface. However, the bubble diameters could not be measured in the present experiment. In previous studies on droplet ejections caused by bubble bursting in seawater and ethanol solutions, the bubble diameters were ~ 10 times larger than the ejected droplet diameters^32,33^. Moreover, the relationship between bubble diameter and droplet diameter is reportedly affected by the surface tension, density, and viscosity of the liquid^34,35^. Therefore, the relationship confirmed in seawater and ethanol solutions cannot be applied to molten tungsten.

Instead, the bubble diameter was estimated by introducing two dimensionless numbers, the Bond number $Bo$ and the Morton number $Mo$. The Bond number is calculated as

2 $$Bo=\frac{\rho g{L}^{2}}{\sigma },$$

where $g$ is the gravitational acceleration and $L$ is the characteristic length. Here, ${Bo}_{\mathrm{b}}$ and ${Bo}_{\mathrm{d}}$ are defined as $Bo$ when the bubble diameter ${d}_{\mathrm{b}}$ and the droplet diameter ${d}_{\mathrm{d}}$ are used as the characteristic length, respectively. The Morton number is given by:

3 $$Mo=\frac{g{\eta }^{4}}{\rho {\sigma }^{3}},$$

where $\eta$ is the viscosity coefficient. When droplets are ejected by bubble bursting at the liquid surface, the Bond and Morton numbers are related as follows^34^:

4 $${Bo}_{d}=\mathrm{A}{Bo}_{b}^\frac{6}{5}{Mo}^{-\frac{1}{3}},$$

where $\mathrm{A}$ is a constant ($8.3\times {10}^{-6}$)^34^. As ${Bo}_{\mathrm{d}}$ and ${Bo}_{\mathrm{b}}$ are functions of the droplet and bubble diameters, respectively, the bubble diameter when bursting at the anode surface immediately before droplet ejection can be estimated from the physical properties of molten tungsten^36^ and the droplet diameter measured by observing the anode appearance.

**Figure 4:**
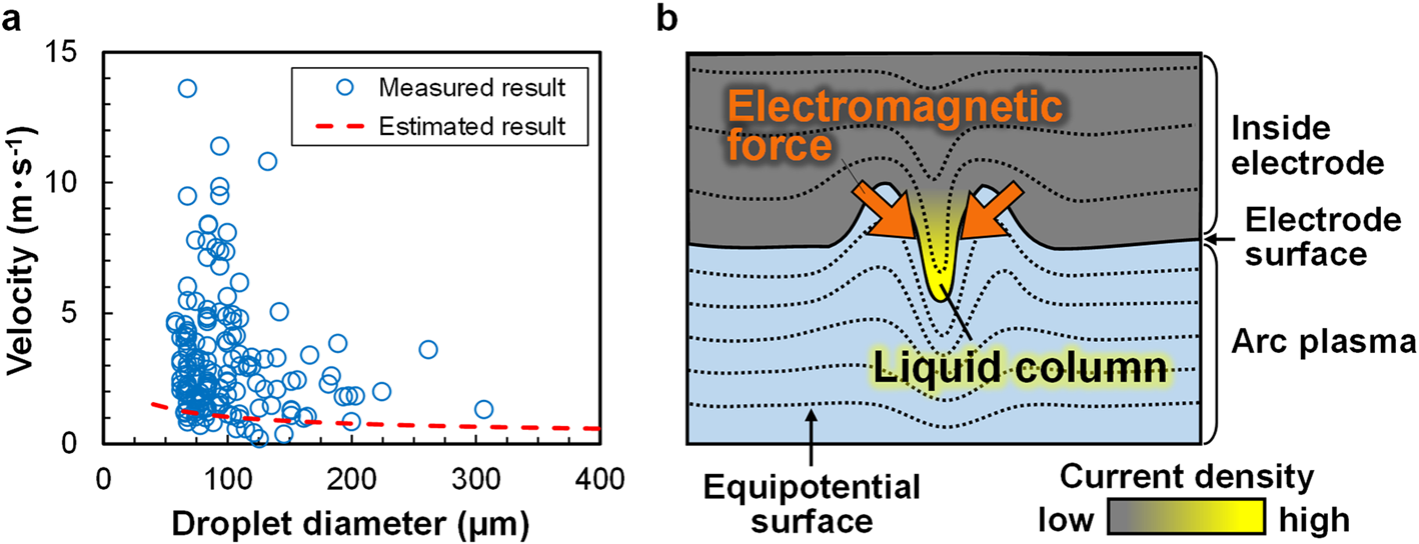
Demonstration of driving force acting on the liquid column: (**a**) Comparison of measured and estimated ejection velocities of droplets with different diameters; (**b**) effect of electromagnetic force on droplet ejection during arc discharge.

Figure 4a compares the droplet-ejection velocity estimated by Eq. (1) and the splashing velocities of droplets with different diameters extracted from anode observations. The measured splashing velocity tended to exceed the theoretical velocity at all droplet diameters. In addition, the measured velocities of relatively small droplets (diameter ≤ 100 µm) reached 10 m s^−1^ or higher, whereas the theoretical velocity was only 1.2 m s^−1^. Thus, the difference between the theoretical and measured results was exaggerated at small droplet diameters.

As the experimental velocities exceeded the theoretical velocities, driving forces other than surface tension likely affected the droplet ejection during arc discharge. Here, the dominant driving force was considered as the electromagnetic force. Figure 4b illustrates the liquid-column growth process on the anode surface during arc discharge. First, a concavity is formed by a bubble bursting at the anode surface. Under the surface tension acting on the rim of the concavity, the flow is driven towards the bottom of the concavity and a liquid column then forms. The elongating liquid column alters the shape of the equipotential surfaces inside and around the column, increasing the current density flowing through the column. The electromagnetic force squeezes the column and pushes it towards the outside of the electrode, promoting the liquid-column growth^37^. Consequently, high-velocity droplets are ejected from the anode surface.

## Discussion

**Figure 5:**
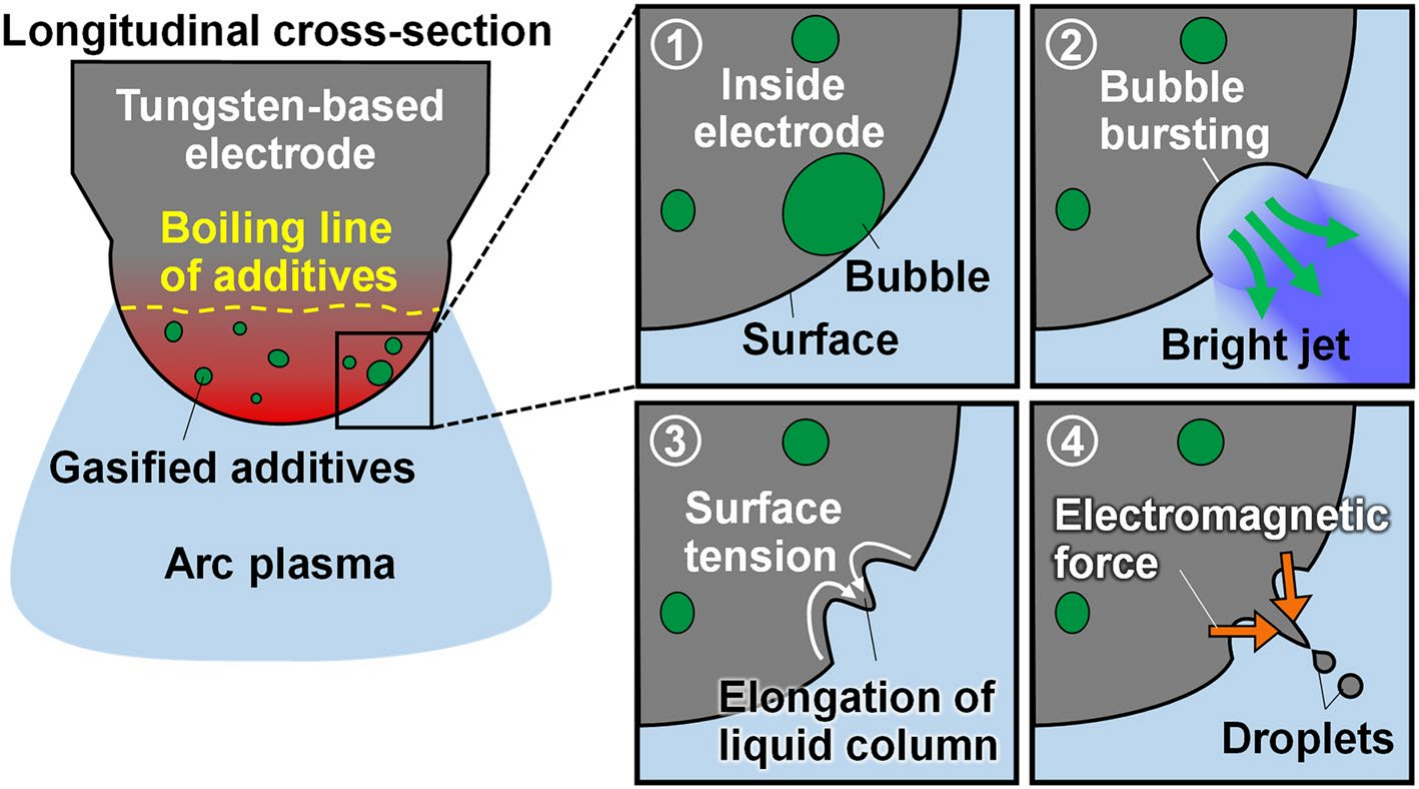
Splashing mechanism from a ceria-tungsten anode during arc discharge.

Figure 5 is a schematic of the splashing mechanism of the ceria-doped tungsten anode, derived from the above experimental findings. When the anode temperature exceeds the boiling point of ceria, the ceria inside the anode gasifies and forms bubbles, which are transported to the anode surface by convection in the molten metal. The bubbles burst, releasing ceria gas into the arc plasma. Within the high-temperature arc plasma, the ceria gas is dissociated and ionised into plasma. Meanwhile, bubble rupture and gas-jet generation depress the molten anode surface. As is often reported in fluid mechanics studies^28–30^, the surface tension acting on the rim of the concavity drives the flow towards the bottom of the concavity, and the rapid concentration of molten metal at the bottom results in the formation of a liquid column. In addition, the elongating liquid column affects the shape of the equipotential surfaces inside and around the column, thus increasing the current density flowing through the column. This activity generates an electromagnetic force that squeezes the liquid column and pushes it towards the outside of the electrode. The column growth is promoted and the tip of the column breaks off and disperses as a high-speed droplet.

Anode splashing in arc discharge is a unique phenomenon caused by a series of processes: melting of the base metal, bubble formation of additives, bubble bursting at the surface, generation of a micro-plasma jet, liquid-column formation due to surface tension, column elongation and breakoff induced by an electromagnetic force, and high-speed droplet ejection. This novel finding will contribute to the stabilisation of arc discharges and assist the exploration and development of emerging technologies such as materials joining, additive manufacturing of three-dimensional printings, and solar power conversion.

## Methods

### Experimental conditions

**Figure 6:**
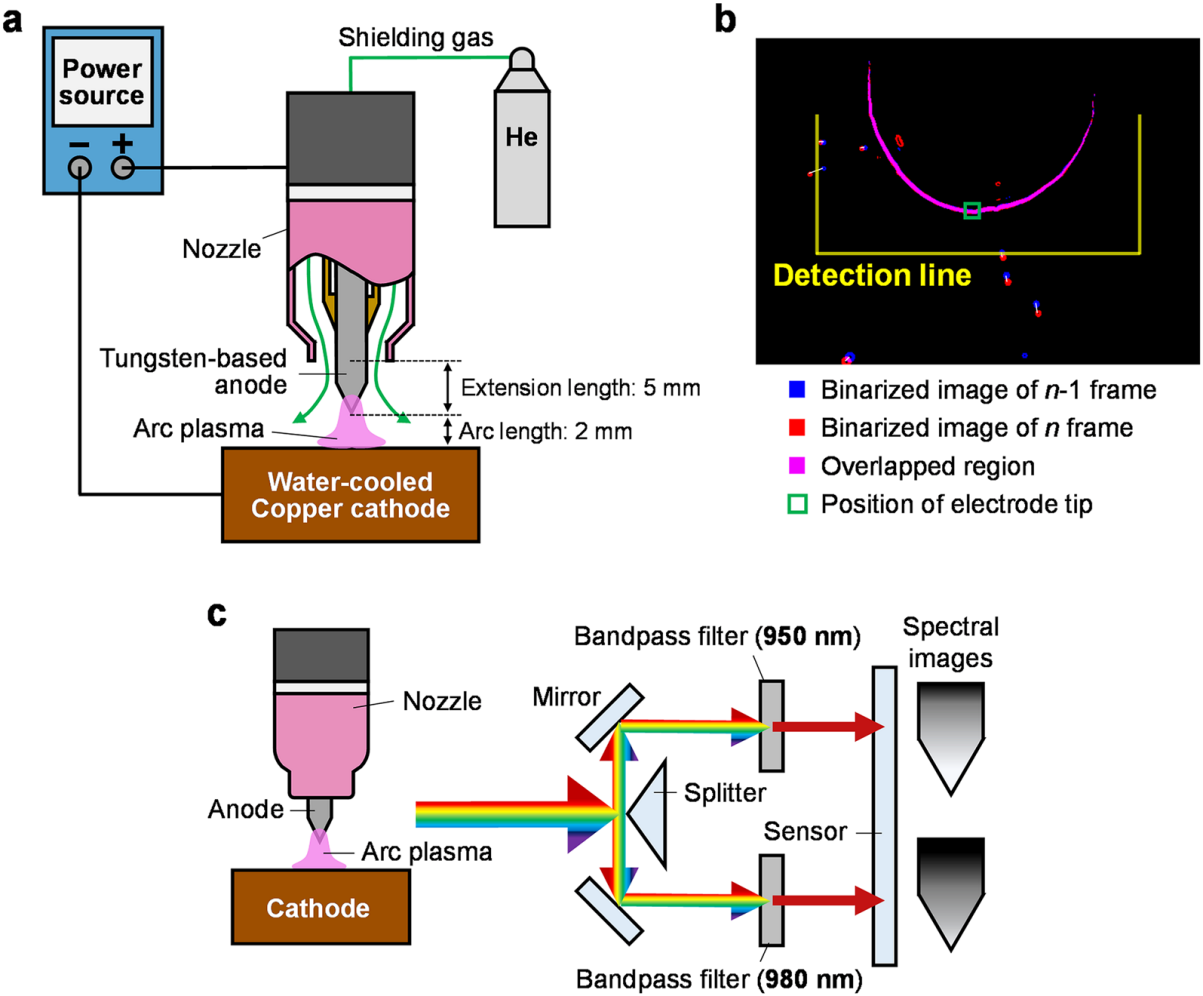
Schematic of the experimental methods: (**a**) system for generating arc discharges; (**b**) measurements of droplet volume and velocity of splashing droplets; (**c**) setup for measuring the anode surface temperature.

Figure 6a is a schematic of the experimental setup for arc discharge. The arc was generated and maintained using a welding power source (DA300P, DAIHEN). The anode material was tungsten with different additives (2 wt% cerium oxide, 2 wt% lanthanum oxide, or 2 wt% thorium oxide). The anode diameter and anode-tip angle prior to arc discharge were 3.2 mm and 60°, respectively. During arc discharge, the upper part of the anode was water-cooled and the nozzle tip was placed 5 mm from the anode tip. A water-cooled copper plate was used as the cathode. The cathode surface and anode tip were separated by 2 mm. During the discharge, a shielding gas of pure helium was flown through the nozzle at 25 L/min. The nozzle diameter was 12.7 mm.

### Observation of the anode appearance

Colour images of the electrode appearance were captured by a high-speed colour camera (MEMRECAM ACS-1 M16, Nac Image Technology). The camera lens was composed of a single focus lens (ED AF MICRO NIKKOR 200 mm 1:4 D, Nikon), a teleconverter (TELEPLUS HDpro 2X DGX, Kenko), and a neutral density filter (ND2, Kenko). The aperture, exposure time, and frame rate were set to f/32, 0.6 µs, and 75,000 frames per second, respectively.

### Visualisation of the splashing trajectory

The splashing trajectories in each image were visualised through image processing using Image J^38,39^. First, edge detection and binarisation were applied to remove the light emission derived from the arc plasma and to clarify the droplets. The binarisation process converts the brightness values in the image to binary data (0 or 1). After identically processing 7500 consecutive frames (total time 0.1 s), the numerical data were summed. Large and small differences in the numerical data reflected the degree of overlap of frames. To visualise the splashing trajectories, the degrees of overlap were represented by colours^40^.

### Analysis of the splashing characteristics

The movement of each droplet was tracked as shown in Fig. 6b. The droplet tracking method measures the droplet size, splashing velocity, and splashing amount. The blue and red parts in Fig. 6b are the results of frames *n*-1 and *n*, respectively, after edge detection and binarisation of the images. The purple part shows the overlapping area in the two binarised images. A single droplet was detected by comparing each droplet detected in frames *n*-1 and *n*. To narrow the number of same-droplet candidates, it was assumed that 1) the diameter of a droplet remains approximately constant during movement over a short time period, and 2) the droplet moves no more than 0.12 mm within one frame. Among the selected candidates, the droplet with the smallest movement of its weighted centre was treated as the same droplet. The white lines in Fig. 6b indicate the movements of the weighted centres of the droplets from frame *n*-1 to frame *n*. The yellow line is the droplet-detection line determined from the anode-tip position (indicated in the green frame). When the weighted centre of a certain droplet moved across the detection line, that droplet was counted as an ejected droplet. The splashing velocity was calculated from the moved distance of the weighted centre at a given time. In addition, the droplets were assumed spherical and the splashing amount was determined from the measured droplet diameter and the density of molten tungsten^25^.

### Surface temperature measurements

The anode surface temperature was measured using two-colour pyrometry^41^. Based on Planck's radiation law, this method calculates the temperature from the ratio of emission intensities from an object obtained at two different wavelengths in a narrow wavelength band. Figure 6c is a schematic of the temperature-measurement device. The incident light from the objective lens was split into two beams by mirrors, passed through two bandpass filters with different wavelengths, and imaged with the charge-coupled-device image sensor of a high-speed camera (MEMRECAM q1v, Nac Image Technology). In this study, the wavelengths of the bandpass filters were 950 and 980 nm.

## Supplementary Information

Supplementary Information.

## Data Availability

The datasets used and analysed during the current study available from the corresponding author on reasonable request.
