# Supplementary material for: Splashing of tungsten-based anode during arc discharge
Source: Sci Rep. 2023 Jul 27;13:12210. doi: 10.1038/s41598-023-39274-4 (PMC10374662; doi:10.1038/s41598-023-39274-4)
Supplement: Supplementary file 1 — Supplementary Information. [file 41598_2023_39274_MOESM1_ESM.pptx]

## Slide 1
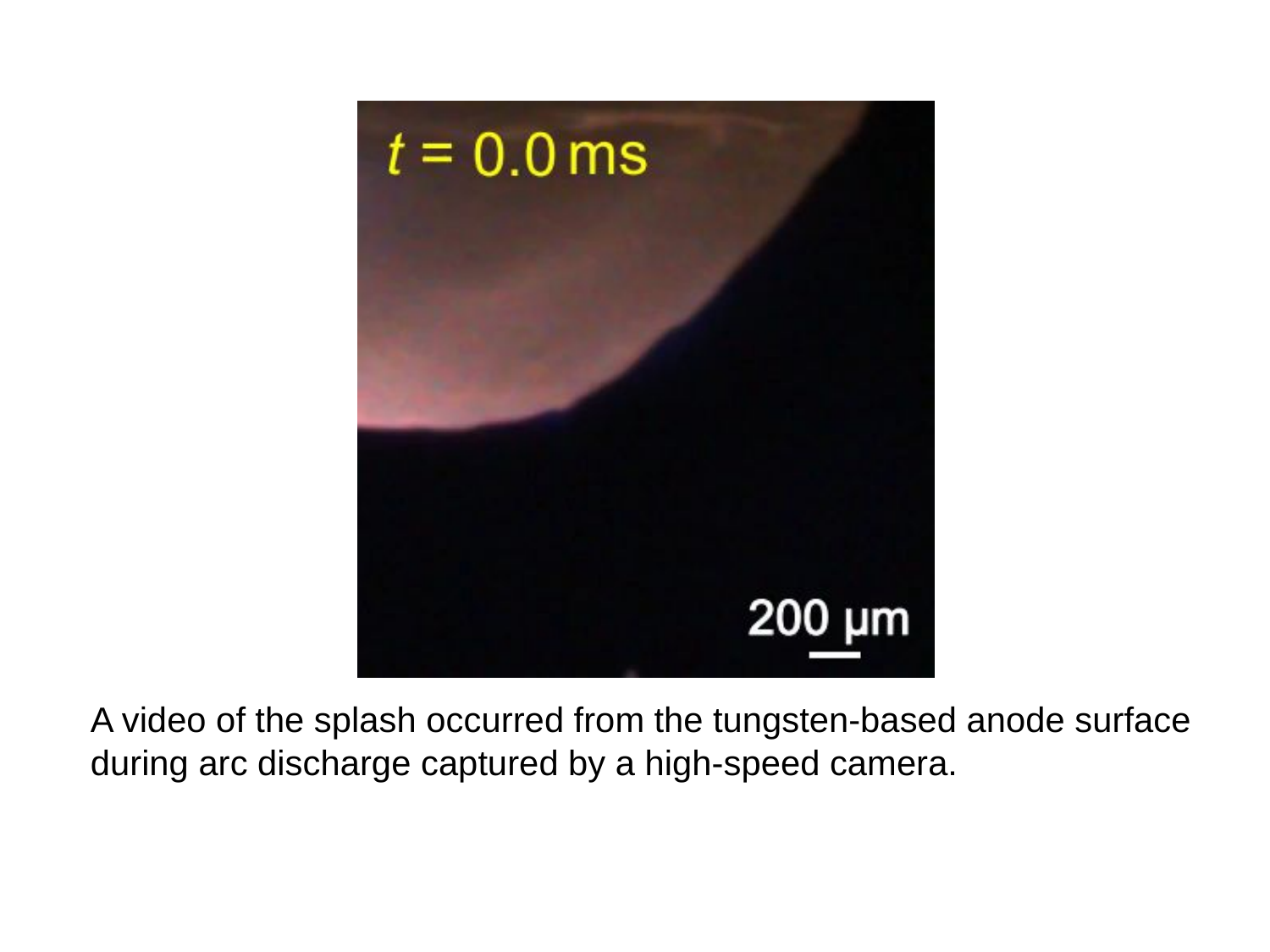

A video of the splash occurred from the tungsten-based anode surface
during arc discharge captured by a high-speed camera.
